# Can Trait Mindfulness Improve Job Satisfaction? The Relationship Between Trait Mindfulness and Job Satisfaction of Preschool Teachers: The Sequential Mediating Effect of Basic Psychological Needs and Positive Emotions

**DOI:** 10.3389/fpsyg.2021.788035

**Published:** 2021-12-13

**Authors:** Zhanmei Song, Baocheng Pan, Youli Wang

**Affiliations:** School of Education, Wenzhou University, Wenzhou, China

**Keywords:** preschool teachers, trait mindfulness, job satisfaction, basic psychological needs, positive emotions

## Abstract

**Objective:** This study aims to explore the relationship between basic psychological needs and positive emotions of preschool teachers between trait mindfulness and job satisfaction.

**Methods:** Three hundred and ninety-eight preschool teachers were tested with mindfulness attention awareness scale, basic psychological needs scale, positive emotion scale, and job satisfaction scale.

**Results:** Preschool teachers trait mindfulness can predict job satisfaction (*β* = 0.265, *p* < 0. 001). Preschool teachers trait mindfulness has an indirect impact on job satisfaction through basic psychological needs (*β* = 0.059, *p* = 0.002), and preschool teachers trait mindfulness has an indirect impact on job satisfaction through positive emotions (*β* = 0.123, *p* < 0. 001). In addition, basic psychological needs and positive emotions play a sequential intermediary role between preschool teachers trait mindfulness and job satisfaction (*β* = 0.017, *p* < 0. 001).

**Conclusion:** Basic psychological needs and positive emotions play a sequential mediating role between preschool teachers trait mindfulness and job satisfaction, and this sequential mediating effect accounts for a high proportion of the total effect.

## Introduction

Teachers’ job satisfaction has become an important topic of concern in the international community, because job dissatisfaction may directly lead to teachers’ resignation (Darling-Hammond, 2003; Liu, 2007; Skaalvik and Skaalvik, 2011). In fact, teacher job satisfaction has been recognized as a decisive factor in the improvement of teaching quality in schools with retained teachers (Huang, 2001). Teaching is a profession with a high attrition rate, and schools struggle to retain talented teachers (Milner and Hoy, 2003). In the United States, up to 25% of new teachers leave the teaching field before the third year, and nearly 40 percent leave the teaching profession within the first 5 years (Hoy and Woolfolk, 1993; Milner and Hoy, 2003; Smith and Ingersoll, 2004). In Britain, far more teachers leave than stay until retirement (Macdonald, 1999). There is also a shortage of high-quality teachers in China (Skaalvik and Skaalvik, 2011). Most distressing of all, there is evidence that many talented teachers leave teaching early (Heyns, 1988).

One of the core problems leading to teacher turnover is teachers’ job satisfaction (Co-investigator, 2002; Coleman, 2012; Nichols, 2018). For teachers with high job satisfaction, there will be less turnover intention; on the contrary, teachers with low job satisfaction will have higher turnover intention. In particular, preschool teachers face higher vocational pressure (Jeon et al., 2019; Kim et al., 2020; Lawrence et al., 2020), need higher emotional investment (Zinsser et al., 2013; Krzewina, 2014) and work investment (Fu, 2015). Therefore, they may have higher job burnout (Clipa and Boghean, 2015; Wells, 2015; Terzic-Supic et al., 2020) and higher turnover intention (Noble and Macfarlane, 2005). Even for some teachers who remain on the job, not having high levels of job satisfaction can lead to inefficiency and burnout, unintentionally harming classrooms and schools (Soetan and Oyetayo, 2019). Therefore, preschool teachers’ job satisfaction has become a key topic that must be paid attention to.

Teachers’ job satisfaction is related to many factors. Caprara et al. (2003), for example, hold that efficacy belief serves as a determinant of teachers’ job satisfaction. Mertler (2001) believes that teacher motivation affects job satisfaction. Sergiovanni (1967) believes that teachers’ job satisfaction is closely related to achievement motivation. All these studies are carried out from the internal factors of teachers themselves. Other studies attempt to study the factors affecting teachers’ job satisfaction from external factors. For example, Lee and Park (2015) research found that organizational justice and commitment of kindergarten teachers had a significant impact on job satisfaction. Hossain et al. (2012) conducted an empirical study on 41 teachers in 10 daycare centers and found that teachers’ job satisfaction was positively correlated with their interaction with children and colleagues, resources, and training. Other studies have shown that teachers’ job satisfaction is related to their leader’s style (Menon, 2014; Falokun, 2017).

However, these studies do not involve the field of metacognition, especially for preschool teachers, the impact of trait mindfulness on job satisfaction is even less. Teacher’s job satisfaction is the teacher’s emotional reaction to his or her job or teaching role (Zembylas and Papanastasiou, 2004; Skaalvik and Skaalvik, 2010). As a kind of emotional reaction, it must be related to the teacher’s own metacognition. Mindfulness has been shown to improve happiness (Hwang et al., 2017; Czerwinski et al., 2020; Tarrasch et al., 2020) and job satisfaction (Hülsheger et al., 2013; Andrews et al., 2014; Vinothkumar et al., 2016). However, few studies have investigated the relationship between preschool teachers trait mindfulness and job satisfaction. Through empirical research, this study explores the relationship between preschool teachers trait mindfulness and their job satisfaction in the field of metacognition and internal factors, which has an important theoretical contribution.

## Literature Review and Theoretical Hypotheses

### Trait Mindfulness and Job Satisfaction

Mindfulness is defined as the subconscious with an observational, non-judgmental stance (Brown and Ryan, 2003; Bishop et al., 2004; Brown et al., 2007; Zeidan et al., 2015). Individuals who are mindful tend to show steady attention, noticing and accepting their immediate responses to thoughts, feelings, and physical stimuli, including their own internal bodily sensations (Khanna and Greeson, 2013). The ability to stay in the moment and be aware of your thoughts and feelings while suspending judgment improves self-regulation and reduces reactivity (Hölzel et al., 2011; Schussler et al., 2016). Mindfulness is actually an individual state of consciousness. Although awareness and attention to current events and experiences are endowed with the characteristics of the human organism, these characteristics can vary considerably from highly lucid and sensitive states to low levels, such as habitual, automatic, unconscious, or dulled thoughts or actions (Wallace, 1999).

At the trait level, mindfulness refers to the cross-situational and relatively stable individual differences in the tendency to be in a state of mindful consciousness (Brown et al., 2007; Grossman, 2011). Research has shown that mindfulness, while varying between individuals, has characteristics with similar characteristics that can be reliably assessed using a number of self-reported measures designed for untrained respondents (Walach et al., 2006; Feldman et al., 2007; Brown et al., 2011). In a growing number of studies, researchers are using these self-reported measures to show a meaningful association between individual naturally occurring trait mindfulness and mental health in non-clinical samples with no experience of meditation or mindfulness training (Weinstein et al., 2009; Niemiec et al., 2010; Bowlin and Baer, 2012; Kiken and Shook, 2012). In the workplace, trait mindfulness is also gaining a lot of attention among organizational scientists (Reb et al., 2014). Some scholars have suggested that mindfulness allows organizations to perform more reliably (Weick et al., 1999; Weick and Sutcliffe, 2006) and perform better in high-speed environments (Dane, 2011). Other studies have shown that trait mindfulness is positively correlated with task performance (Reb et al., 2014; Jahanzeb et al., 2020). Trait mindfulness also helps employees self-regulate their behavior (Lyvers et al., 2014; Kadziolka et al., 2016) to enhance happiness (Reb et al., 2014; Jin et al., 2020; Liu et al., 2021) and higher task performance (Glomb et al., 2018).

According to affective event theory, trait mindfulness may be positively correlated with job satisfaction (Weiss and Cropanzano, 1996). Work events are a proximate source of employees emotional reactions, which in turn predict job satisfaction (Hülsheger et al., 2013). School teaching is one of the professions that requires particularly high levels of emotion regulation skills, which are necessary to successfully manage challenging student behavior and deal with their own emotional reactions (Skinner and Beers, 2016). Studies have shown a significant link between mindfulness skills, teacher occupational health and well-being, and classroom practice among preschool teachers (Jennings, 2015). In addition, trait mindfulness fosters self-determined behavior that is aligned with an individual needs and values (Deci and Ryan, 1985; Brown and Ryan, 2003). Trait mindfulness may have a positive effect on job satisfaction by promoting self-determination behavior (Glomb et al., 2011).

Based on this, the following hypotheses are proposed in this study:

*H1:* Trait mindfulness of preschool teachers is positively correlated with job satisfaction.

### Trait Mindfulness, Basic Psychological Needs, and Job Satisfaction

An integral part of self-determination theory is the concept of three basic psychological needs (Deci and Ryan, 2000; Ryan and Deci, 2000), namely, autonomy, competence, and relevance. Those whose basic psychological needs are met tend to have higher intrinsic motivation (Klassen et al., 2012). The relationship between the satisfaction of basic needs and happiness has been widely demonstrated in general and specific areas of life and in general social contexts (Piccolo et al., 2005; Demir and Özdemir, 2010; Sapmaz et al., 2012). In the workplace, meeting basic needs has also been shown to be associated with successful job performance by positive psychological regulation (Baard et al., 2004), enhancing positive emotions, reducing negative emotions (Tong et al., 2009), and increasing job engagement (Van Den Broeck et al., 2008). In the field of education, research has shown that the satisfaction of teachers basic needs is associated with both positive and negative aspects of well-being and classroom outcomes, such as job commitment and burnout (Abós Catalán et al., 2018), work enthusiasm (Aldrup et al., 2017), and teaching behavior (Korthagen and Evelein, 2016).

Studies have shown that mindfulness may enhance a personal receptivity to events and experiences (Brown and Ryan, 2012). When people are aware of their own internal basic psychological needs, self-involvement is less likely to dominate the internal interactions of the individual mind. In this situation, people are more likely to act autonomously (Scott Rigby et al., 2014; Schultz et al., 2015). In addition, people who are focused on the present moment are likely to be fully aware of both the internal and external world. This provides a more autonomous, less controlling, or defensive base state in which the individual can fully participate (Chang et al., 2015). At this point, the individual is more able to view feedback as information rather than control because the individual is less likely to feel self-involved in feedback. Therefore, mindfulness can enhance autonomy and competence through feedback (Scott Rigby et al., 2014; Schultz et al., 2015). At the same time, in the field of teaching, such attentional limitations and motivational selective bias may hinder the receptivity to positive teaching behaviors of teachers, and these events and experiences can satisfy teachers basic psychological needs (Li et al., 2019).

Meeting basic psychological needs is seen as a key determinant of experiencing the good life (Deci and Ryan, 2000). Experiencing the good life is one of the meanings of teachers’ work. Many teachers find personal satisfaction and happiness in their work (Klassen and Chiu, 2010). According to Locke (1976), job satisfaction can be defined as “a pleasant or positive emotional state resulting from an evaluation of one’s work or work experience.” Pietsch et al. (2019) believes that job satisfaction is a personal multidimensional psychological response to work, including cognitive (evaluation), emotional (or emotional), and behavioral components. Studies show that when the basic psychological needs of individuals are satisfied, there will be higher job satisfaction (Arshadia, 2010). In particular, a study of teachers shows that teachers’ job satisfaction is significantly correlated with basic psychological needs (Wininger and Birkholz, 2013).

In conclusion, this study proposes hypotheses:

*H2:* Basic psychological needs mediate the relationship between trait mindfulness and job satisfaction.

*H2a:* Trait mindfulness is positively correlated with basic psychological needs.

*H2b:* Basic psychological needs are positively correlated with job satisfaction.

### Trait Mindfulness, Positive Emotion, and Job Satisfaction

Emotions arise when a person notices a situation, evaluates it as related to his or her needs, values or goals, and responds to the situation with loosely coupled changes in subjective experience, behavior, and physiological fields (Scherer, 2000). In previous studies, negative emotions have been widely concerned, but the research on positive emotions has increased exponentially in the past two decades (Quoidbach et al., 2015). More and more studies show that positive emotions help people deal with stress (Wichers et al., 2008; Avey et al., 2011; Curtin, 2016) and psychological capital (Azab et al., 2018; Carmona-Halty et al., 2021). Positive emotions have always been regarded as an important part of mental health (Taylor and Brown, 1988).

At the same time, researchers found that positive emotions are positively correlated with the quality of social interaction (Sroufe et al., 1985; Denham et al., 1990; McDowell and Parke, 2005), and inducing positive emotions can promote prosocial behavior (Rosenhan et al., 1974). In school settings, research evidence has shown that teachers’ emotional expression will have an important impact on students. Teachers express anger at students’ failure due to lack of effort (Graham, 1984; Graham, 1990; Clark, 1997; Clark and Artiles, 2000), and sympathize or pity for students’ failure due to lack of ability. The emotional expression of these teachers will affect students’ attribution to the causes of success and failure (Graham, 1984; Weiner, 2000). If teachers can control their emotions and use positive emotions to express their expectations and requirements, it will lead to students positive attribution.

Emotion regulation and emotion control have been repeatedly proposed as the central mechanism in the theoretical research of mindfulness (Roemer and Orsillo, 2003; Bishop et al., 2004; Hayes and Feldman, 2004; Glomb et al., 2011). The basic purpose of mindfulness training is to improve the trainees positive emotion and produce ideal results. Studies have shown that trait mindfulness can predict lower levels of daily negative effects (Brown and Ryan, 2003; Weinstein et al., 2009), more effective emotional regulation (Baer et al., 2006), and higher happiness (Weinstein et al., 2009). The laboratory research conclusion also shows that mindfulness is related to reducing the subjective or physiological response to emotional stressors (Arch and Craske, 2006; Wolgast et al., 2011). Keng and Tong (2016) empirical research also shows that there is a negative correlation between trait mindfulness and negative emotional instability. People with higher trait mindfulness have higher concentration, which makes them less inclined to use maladaptive strategies as coping mechanisms (Hamilton et al., 2006), which helps to weaken their response to daily stressors.

Job satisfaction is closely related to emotion. First of all, from the definition of job satisfaction itself, job satisfaction is considered to be people positive or negative evaluation and judgment of work (Weiss, 2002). More specifically, job satisfaction is a pleasant or positive emotional state due to the evaluation of work (Locke, 1976). Secondly, the research shows that positive emotion and negative emotion can explain the change of employee job satisfaction. Employees who tend to be happy (positive emotions) are more likely to have higher job satisfaction than employees who tend to experience discomfort (negative emotions; Staw and Ross, 1985; Staw et al., 1986; Duffy et al., 1998).

According to the expansion and construction theory of positive emotion (Fredrickson, 1998), positive emotion is the adaptive function of evolution to establish lasting resources. It will produce novel and extensive ideas and actions. This state will have a positive impact on job satisfaction. For schools, teachers are one of the important teachers in school organizations (Hattie, 2003). Especially for teachers in the classroom environment, “emotional contagion” effect is particularly important. Frequent positive emotions are transferred from teachers to students (Frenzel et al., 2009). Positive emotion is associated with internal motivation and the ideal image of “enthusiastic teacher.” Under this positive emotion, the teacher is good at motivating his or her students to maximize their potential (Kunter and Holzberger, 2014).

Therefore, we propose hypotheses:

*H3:* Positive emotion plays an intermediary role between trait mindfulness and job satisfaction.

*H3a:* Trait mindfulness is positively correlated with positive emotions.

*H3b:* Positive emotion is positively correlated with job satisfaction.

### Basic Psychological Needs and Positive Emotions

The satisfaction of basic needs is closely related to positive psychological adjustment (Baard et al., 2004), enhancing positive emotions and reducing negative emotions (Tong et al., 2009). In the school education environment, the satisfaction of teachers basic needs can not only affect students motivation and academic performance (Reeve et al., 1999), but also promote students perception of autonomy support in order to obtain a better learning experience (Jang et al., 2009).

At the same time, Klassen et al. (2012) research shows that there is also a close relationship between the satisfaction of teachers basic psychological needs and their work input and emotion in the educational workplace. The research results of Katz and Shahar (2015) also show that teachers who teach out of interest and fun and teachers who pay attention to their work (autonomous machine) tend to think that autonomous students are more beneficial to their learning.

Teachers’ positive and negative emotions are very important to educational results, because they affect teachers’ efficiency by affecting students cognition, emotion, and motivation (Sutton and Wheatley, 2003). According to the self-determination theory (Ryan and Deci, 2000), teachers basic psychological needs are met, often have internal motivation, and produce positive emotions.

In conclusion, we put forward the following hypotheses:

*H4:* Basic psychological needs are positively correlated with positive emotions.

*H5:* Basic psychological needs and positive emotions sequentially mediate the relationship between trait mindfulness and job satisfaction.

**Figure 1:**
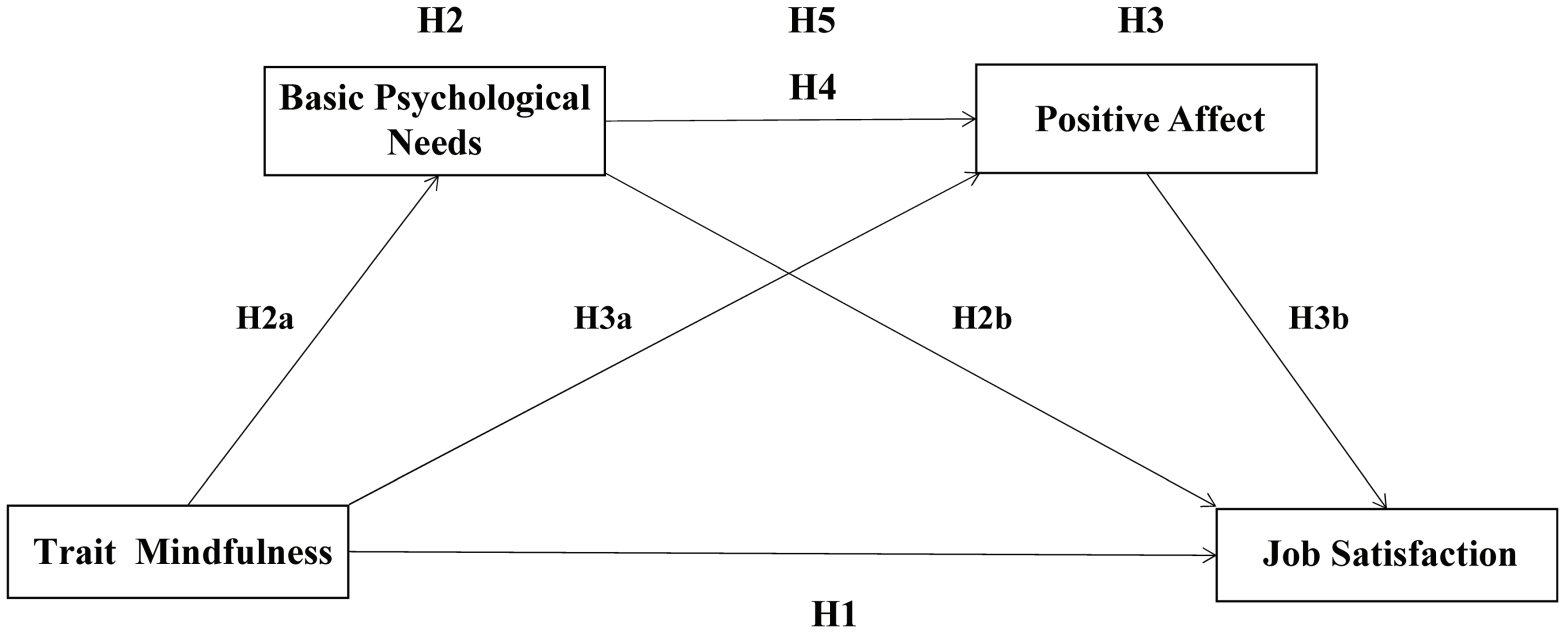
Theoretical hypotheses.

Basic psychological needs and positive emotions sequentially mediate the relationship between trait mindfulness and job satisfaction, as shown in Figure 1. In addition, the conceptual model also puts forward the assumptions in the study.

## Materials and Methods

### Participants

In this study, a convenient random sampling method was used to sample kindergartens in Jinan City, Shandong Province, China. The kindergarten teachers participating in this survey are all full-time in-service teachers, and a total of 398 preschool teachers participated in the survey. Among them, 33 were male teachers, accounting for 8.30%, and 365 were female teachers, accounting for 91.70% (see Table 1 for details). The formal implementation is in charge of preschool education interns who have received unified training. After communicating and agreeing with the person in charge of the kindergarten, the questionnaire was distributed online for collection. Each test adopts uniform guidelines and emphasizes the confidentiality of the survey to ensure the validity and authenticity of the questionnaire. A total of 445 questionnaires were distributed and 420 were actually returned. For questionnaires with accurate data, we use the direct elimination method. In the end, 398 valid questionnaires were recovered, with an effective recovery rate of 89.44%. Ethical review and approval were waived for this study by the Research Ethics Committee of the Wenzhou University according to the guidelines of the Declaration of Helsinki, as the study involving questionnaire survey did not involve personal privacy issues, yet issues on psychological or physical harms to participants.

**Table 1:** Social demographic features of participants (*N* = 398).

Variables		Percentages
Gender	Male	8.30%
	Female	91.70%
Age	25–27	83.92%
	28–30	6.78%
	31–33	5.78%
	34–36	3.52%
Educational Background	Junior college	72.10%
	Undergraduate course	27.90%
Kindergarten Site	Rural kindergarten	29.90%
	Township kindergarten	35.20%
	City kindergarten	34.90%
Kindergarten Nature	Private kindergarten	51.30%
	Public kindergarten	48.70%

### Measures

#### Mindfulness Attention Awareness Scale

Trait mindfulness adopts the mindfulness attention awareness scale compiled by Brown and Ryan (2003), which contains 15 questions. This scale has been proved to have good reliability and validity in Chinese population (Black et al., 2012). A sample item was “I could be experiencing some emotion and not be conscious of it until sometime later.” The instruction requires the subjects to select the most suitable description level in each item according to the actual situation in the recent week (including the day). Survey questionnaire was measured with 6-point Likert scale for measuring (1 = almost always and 6 = almost never). High scores reflect a high level of awareness and attention to the present in an individual daily life. In this study, Cronbach’s α value was 0.947.

#### Basic Psychological Needs Scale

The basic psychological needs scale was compiled by Deci and Ryan (2000). This scale has proved to have good reliability and validity in China (Tian et al. 2016). There are 21 items in the scale, of which 9 items are reverse scoring questions, including capacity needs (6 items), relationship needs (8 items), and autonomy needs (7 items). These projects include the statement: “I often feel weak.” The scale has seven grades (1 = totally disagree and 7 = totally agree). In this study, Cronbach’s α was 0.957, and the Cronbach’s α value of three dimensions was 0.898, 0.887, and 0.910, respectively.

#### Positive Emotion Scale

This study used the positive emotion subscale in the positive negative emotion scale compiled by Watson et al. (1988). The scale contains 10 questions. This scale has been proved to have good reliability and validity in China and is widely used (Huang et al., 2003). The scale consists of many words describing different feelings and emotions. Subjects were asked to read each item and then point out to what extent this feeling appeared. These feelings include “interested,” “excited,” etc. Survey questionnaire was measured with 5-point Likert scale for measuring (1 = almost none and 5 = extremely many). In this study, Cronbach’s α was 0.933.

#### Job Satisfaction Scale

The measurement of job satisfaction adopts the job satisfaction scale compiled by Brayfield and Rothe (1951), which contains five questions. This scale has proved to have good reliability and validity in China. These items include the statement: “My job is like a hobby to me.” Survey scale was measured with 7-point Likert scale for measuring (1 = totally disagree and 7 = totally agree). The higher the score, the higher the individual job satisfaction. In this survey, Cronbach’s α of the scale was 0.831.

#### Statistical Methods and Analysis Ideas

In this study, SPSS 22.0 and Mplus version 8.3 were used for data analysis. SPSS was mainly used for data sorting, descriptive statistical analysis, etc. Mplus is mainly used for model inspection. Participants who lack descriptive data or many data points are processed by list deletion when running the analysis. In the analysis, teachers’ gender, age, education level, the site, and nature of kindergartens are taken as control variables. Gender was dummy coded (0 = male and 1 = female).

## Results

### Test of Common Method Deviation

Using Harman single factor test, 6 factors with characteristic roots greater than 1 were obtained. The interpretation rate of the first factor is 34.446%, which is less than the critical value of 40% (Podsakoff et al., 2003), indicating that there is no obvious common method deviation in this study.

### Descriptive Statistical Analysis

In factor analysis, randomly select 1/2 of the sample (*N* = 199) from the overall data for exploratory factor analysis, and then use the other 1/2 of the samples (*N* = 199) to pass confirmatory factor analysis. Exploratory factor analysis was carried out to uncover the structure of the variables. Exploratory factor analysis convergence validity measures the extent to which the factors of a single structure are consistent. In this study, the convergence validity was evaluated using compound reliability and mean variance interpretation. Using these measures, the combined reliability (CR) of all structures should be higher than 0.6 and the average economic value (AVE) should be higher than 0.5 (Fornell and Larcker, 1981).

As shown in Table 2, the combined reliability of relative variable ranges from 0.833 to 0.951, while the average economic value of relative variable ranges from 0.503 to 0.585. These results reveal that the research variables are in the acceptable range.

**Table 2:** Reliability, validity statistics, and correlations.

Variable	Item Reliability	Composite Reliability		Convergent Validity		Discriminate validity	
	STD. LOADING	CR	AVE	TM	BPN	PA	JS
TM	0.543–0.816	0.951	0.564	**0.751**			
BPN	0.624–0.826	0.918	0.504	0.382	**0.71**		
PA	0.686–0.850	0.933	0.585	0.595	0.385	**0.765**	
JS	0.601–0.821	0.833	0.503	0.522	0.432	0.57	**0.709**

The diagonal in bold is the square root of AVE, and the lower triangle is the Pearson correlation of the dimension. TM, Trait Mindfulness; BPN, Basic Psychological Needs; PA, Positive Affect; and JS, Job Satisfaction.

Confirmatory factor analysis is used to evaluate the reliability and validity of the overall measurement model and the evaluation structure. In order to evaluate the validity of the measurement model, the discriminant and convergence validity were evaluated. Fornell and Larcker’s (1981) methods were used to evaluate the discriminant validity. Using this method, the mean variance of each study structure should be higher than the square correlation between that structure and any other structure. As shown in Table 2, the measurement model proves satisfactory discriminant validity. The diagonal elements in Table 2 (in bold) are the square multiple correlations between the variables studied. As shown in the table, AVE ranges from 0.503 to 0.585, while diagonal values range from 0.709 to 0.765, indicating that diagonal variables are higher than various AVE values, indicating that all structures in this study have sufficient discriminant validity.

Table 3 lists the Pearson correlation coefficients between the main variables and their dimensions. It can be seen from Table 1 that trait mindfulness has a significant positive correlation with basic psychological needs, positive emotions, and job satisfaction.

**Table 3:** Means, standard deviations, and correlations of the major study variables.

Variable	*M*	*SD*	1	2	3	4	5	6	7	8	9
1. Gender	0.92	0.28	1								
2. Age	27.10	2.39	−0.057	1							
3. Educational Background	1.28	0.45	0.126^*^	−0.058	1						
4. Kindergarten Site	2.05	0.81	−0.049	−0.068	0.407^**^	1					
5. Kindergarten Nature	1.49	0.50	0.129^**^	−0.031	0.335^**^	0.371^**^	1				
6. TM	3.84	1.14	−0.079	0.05	0.019	0.207^**^	−0.086	1			
7. BPN	3.99	1.19	0.019	0.009	0.075	0.067	0.024	0.368^**^	1		
8. PA	3.02	1.00	−0.052	0.028	−0.005	0.052	−0.044	0.564^**^	0.361^**^	1	
9. JS	4.41	1.07	0.01	−0.026	−0.038	−0.011	−0.024	0.474^**^	0.388^**^	0.512^**^	1

Gender is the dummy variable (0 = male and 1 = female).

*N* = 398.

* *p* < 0.05;

** *p* < 0.01.

According to Tsui et al. (1995), the critical value of correlation level of serious multicollinearity problem generally exceeds 0.75, the correlation coefficient of each variable in this study does not exceed 0.75, and there is no serious multicollinearity problem among main variables.

### Model Inspection

Mplus version 8.3 was used to fit the chain mediation model. The model fitting index was ML *χ*^2^ = 906.77, df = 639, *χ*^2^/df = 1.419, CFI = 0.969, TFI = 0.966, RMSEA = 0.032, SRMR = 0.045. All indexes are in an acceptable range, and the model is ideal. See Table 4.

**Table 4:** Fit indices of the model.

Fit indices	Recommended threshold	Scores	Remarks
ML *χ*^2^	–	906.77	–
Df	–	639	–
*χ*^2^/df	1 < *χ*^2^/df < 3	1.419	Acceptable
CFI	> 0.9	0.969	Acceptable
TLI	> 0.9	0.966	Acceptable
RMSEA	< 0.08	0.032	Acceptable
SRMR	< 0.08	0.045	Acceptable

### The Significance Test of Mediating Effect

On the basis of good model fitting, the Bootstrap program of Mplus was used to repeat the sample for 5,000 times. The results show that the path coefficients between trait mindfulness and basic psychological needs, positive emotions, and job satisfaction are significant, and trait mindfulness can directly predict job satisfaction (*β* = 0.265, *p* < 0. 001), supporting H1. Trait mindfulness can measure basic psychological needs (*β* = 0.406, *p* < 0. 001), supporting H2a. Basic psychological needs can predict job satisfaction (*β* = 0.210, *p* = 0. 001), supporting H2b. Trait mindfulness can predict positive emotions (*β* = 0.535, *p* < 0. 001), supporting H3a. Positive emotions can predict job satisfaction (*β* = 0.333, *p* < 0. 001), supporting H3b. There is a positive correlation between basic psychological needs and positive emotions (*β* = 0.181, *p* < 0. 001), supporting H4. See Table 5.

**Table 5:** The direct effect of the research paths and research model hypothesis analysis.

DV	IV	Std. Est.	*SE*	Est./*SE*	Value of *P*	*R* ^2^	Hypo and Path	Remarks
JS	TM	0.265	0.062	4.275	^***^	0.457	H1:TM → JS	Support
	BPN	0.210	0.061	3.443	0.001		H2b:BPN → JS	Support
	PA	0.333	0.068	4.879	^***^		H3b:PA → JS	Support
BPN	TM	0.406	0.051	7.926	^***^	0.177	H2a:BPN → BPN	Support
PA	TM	0.535	0.045	11.815	^***^	0.403	H3a:TM → PA	Support
	BPN	0.181	0.045	4.058	^***^		H4:BPN → PA	Support

*** *p <* 0.001.

Table 6 shows the indirect impact of the study path. Basic psychological needs play a mediating role between trait mindfulness and job satisfaction (*β* = 0.059, *p* = 0.002), the confidence interval was 95% (0.026–0.099), excluding 0, supporting H2, and the mediating effect accounted for 15.45%.

**Table 6:** The indirect effect of the research paths.

Path	Std. Est.	*SE*	Est./*SE*	Value of *P*	Boot LLCI	Boot ULCI	The proportion of the effect
H2:TM → BPN → JS	0.059	0.019	3.167	0.002	0.026	0.099	15.45%
H3:TM → PA → JS	0.123	0.03	4.037	^***^	0.070	0.190	32.20%
H5:TM → BPN → PA → JS	0.017	0.006	2.74	0.006	0.008	0.033	4.45%
TOTALIND	0.199	0.036	5.455	^***^	0.133	0.276	52.09%
TOTAL	0.382	0.044	8.667	^***^	0.302	0.474	100.00%

*** *p* < 0.001.

Positive emotions play a mediating role between trait mindfulness and job satisfaction (*β* = 0.123, *p* < 0.001), the confidence interval was 95% (0.070–0.190) excluding 0, supporting H3, and the mediating effect accounted for 32.20%.

**Figure 2:**
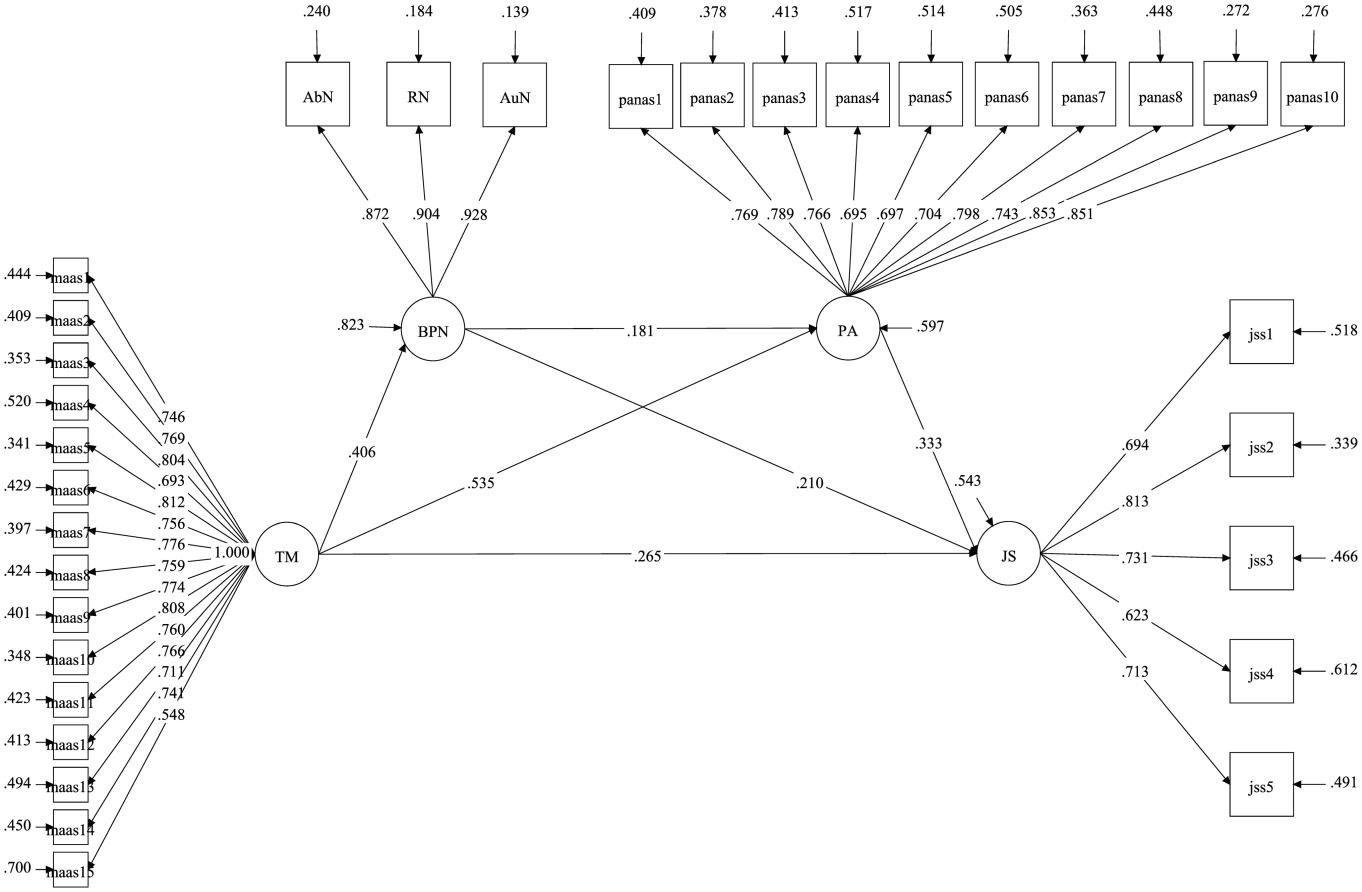
Structural equation.

Basic psychological needs and positive emotions play a sequential mediating role between trait mindfulness and job satisfaction (*β* = 0.017, *p* < 0.001), 95% confidence interval [0.008–0.033], excluding 0, supports H5, and the mediating effect accounts for 4.45%. See Figure 2.

## Discussion

This study discusses the influencing factors of preschool teachers’ job satisfaction from the field of metacognition. The results support the previous research that job satisfaction is positively correlated with the satisfaction of basic psychological needs (Collie et al., 2015; Unanue et al., 2017) and positive emotions (Ziegler et al., 2012; Todorova et al., 2014). At the same time, the results of this study show that for preschool teachers, their job satisfaction is not only related to basic psychological needs and positive emotions, but also closely related to trait mindfulness. The mediating effect of trait mindfulness on job satisfaction through the sequential mediating effect of basic psychological needs and positive emotions accounts for 65.96% of the total effect, which is a very high proportion, indicating that preschool teachers trait mindfulness affects job satisfaction to a great extent. Compared with previous studies, this study has important theoretical contributions.

In addition, the research on preschool teachers trait mindfulness and job satisfaction can provide some suggestions for kindergarten education practice and management practice. Firstly, kindergartens or education management departments should improve the mindfulness level of preschool teachers by adopting mindfulness training and offering mindfulness training courses and providing social support systems. Preschool teachers are facing a high professional pressure and high emotional environment. Working in this working environment with great psychological pressure, preschool teachers must have good emotional management ability and psychological tolerance. Through mindfulness training, improving preschool teachers mental health level and their ability to accept and tolerate children’s challenging and wrong sexual behaviors, guiding preschool teachers to look at problems from a positive perspective, and providing preschool teachers with a strong social support system will effectively improve teachers job satisfaction and job happiness.

Secondly, the basic psychological needs of preschool teachers should be met. In terms of curriculum implementation and work, managers should give preschool teachers autonomy. The authorization of managers for autonomy will improve the basic psychological needs of preschool teachers to a certain extent. The leaders of early education should strengthen the trust in the competence of preschool teachers and let preschool teachers have a sense of competence. At the same time, the leaders of early education should create a good working environment for preschool teachers, which make preschool teachers have a sense of belonging. These measures will improve preschool teachers job satisfaction to a great extent.

Finally, we should let preschool teachers have positive emotions, guide preschool teachers to make positive attribution, and create a good positive psychological environment for them, so as to improve preschool teachers positive emotions. Kindergartens should establish a strong emotional support system to create an environment conducive to releasing and eliminating emotions. At the same time, kindergarten leaders encourage preschool teachers to carry out internal self-dialog and positive psychological suggestion.

## Limitations and Future Research Directions

Firstly, the data of this study are from self-report, and future research may consider using more objective indicators. Secondly, the method used in this study is horizontal and cannot reflect the long-term performance of the mechanism studied in this study. Especially, in an emergency context due to the COVID-19 pandemic which is having a relevant impact on the job satisfaction of kindergarten teachers. The future research should take into account the job satisfaction of kindergarten teachers during the COVID-19 pandemic. Thirdly, the study includes two mediating factors, but there should be more mediating factors in the impact of trait mindfulness on job satisfaction, such as the role of psychological elasticity, subjective well-being, and emotional intelligence in this process. Finally, this study only studies the impact of preschool teachers individual level. Future research can consider the impact of organizational factors and leadership factors on preschool teachers job satisfaction, and can carry out cross-level research.

## Conclusion

The results of this study show that trait mindfulness is positively correlated with job satisfaction. Trait mindfulness can affect job satisfaction not only through basic psychological needs, but also through positive emotions. In addition, the most important finding of this study is that basic psychological needs and positive emotions play a sequential intermediary role between preschool teachers trait mindfulness and job satisfaction. Moreover, this sequential mediating effect accounts for a high proportion of the total effect. We believe these findings will help enrich the literature on preschool teachers job satisfaction and kindergarten management practice.

## Data Availability Statement

The raw data supporting the conclusions of this article will be made available by the authors, without undue reservation.

## Ethics Statement

The studies involving human participants were reviewed and approved by the Research Ethics Committee of the Wenzhou University. The patients/participants provided their written informed consent to participate in this study.

## Author Contributions

BP designed, prepared, and carried out the data collection process, and written the article. ZS revised the section of the analysis and discussion and corrected the entire manuscript. YW analyzed and verified the data in this article. All authors contributed to the article and approved the submitted version.

## Funding

This work was supported by the second batch of teaching reform research projects of higher education in Zhejiang Province during the “13th Five-Year Plan” (project number: jg20190401). This project is supported by the Graduate Research and Innovation Fund of Wenzhou University.

## Conflict of Interest

The authors declare that the research was conducted in the absence of any commercial or financial relationships that could be construed as a potential conflict of interest.

## Publisher’s Note

All claims expressed in this article are solely those of the authors and do not necessarily represent those of their affiliated organizations, or those of the publisher, the editors and the reviewers. Any product that may be evaluated in this article, or claim that may be made by its manufacturer, is not guaranteed or endorsed by the publisher.
